# Pleural Epithelioid Hemangioendothelioma (EHE): A Case Report

**DOI:** 10.7759/cureus.41308

**Published:** 2023-07-03

**Authors:** Vikas Pathak, Christopher Walker

**Affiliations:** 1 Pulmonary and Critical Care, Virginia Institute of Lung Diseases, Yorktown, USA; 2 Internal Medicine, George Washington University School of Medicine and Health Sciences, Washington DC, USA

**Keywords:** pleural thickening, epithelioid hemangioendothelioma, malignancy, pleural effusion, pleura

## Abstract

Pleural epithelioid hemangioendothelioma (EHE) is a rare malignancy of vascular origin. It can affect various organs; pleural involvement is rare and lack of suspicion leads to delay in diagnosis. We present a case of pleural EHE with metastasis to lung parenchyma that presents with chest pain and dyspnea. Chest imaging showed loculated pleural effusion, pleural thickening, mediastinal lymphadenopathy, and pulmonary nodules.

## Introduction

Pleural effusion is one of the most common diseases involving pleural space. The etiology of pleural effusion is varied and its presence could be due to benign or malignant conditions affecting the pleura. Epithelioid hemangioendothelioma (EHE) of the pleura is a rare subtype of an uncommon malignancy and proves a diagnostic challenge. It reportedly involves bone, meninges, soft tissue, gastrointestinal tract, mediastinum, spleen, breast, testicle, and skin. Pleural involvement is rare [1].

Herein is described a patient who presented with pleural effusion and pleural thickening, who underwent multiple thoracentesis with no clear diagnosis. Eventually after thoracoscopy and pleural biopsy, she was diagnosed with pleural EHE. 

## Case presentation

**Figure 1:**
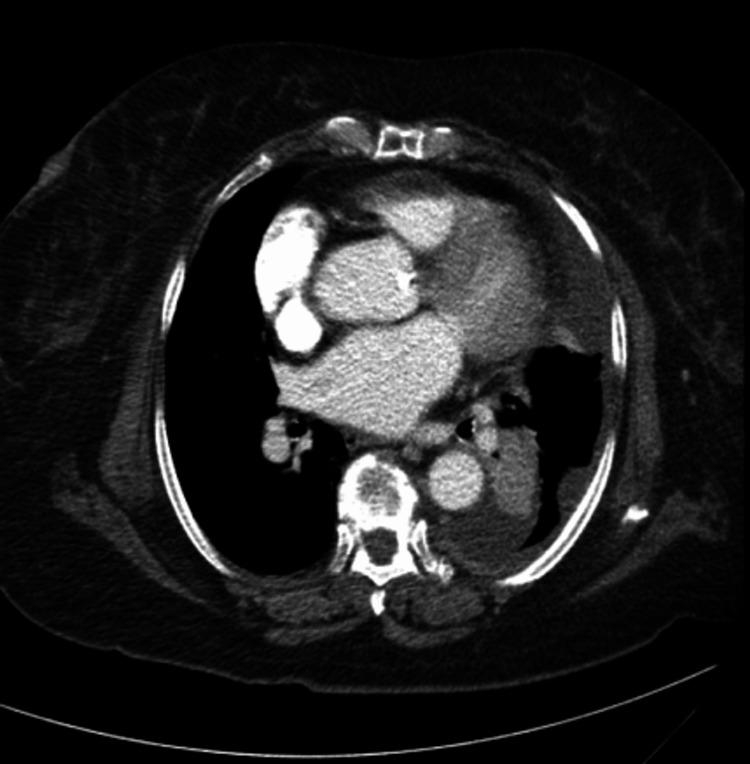
CT scan showing left-sided loculated pleural effusion and pleural thickening.

**Figure 2:**
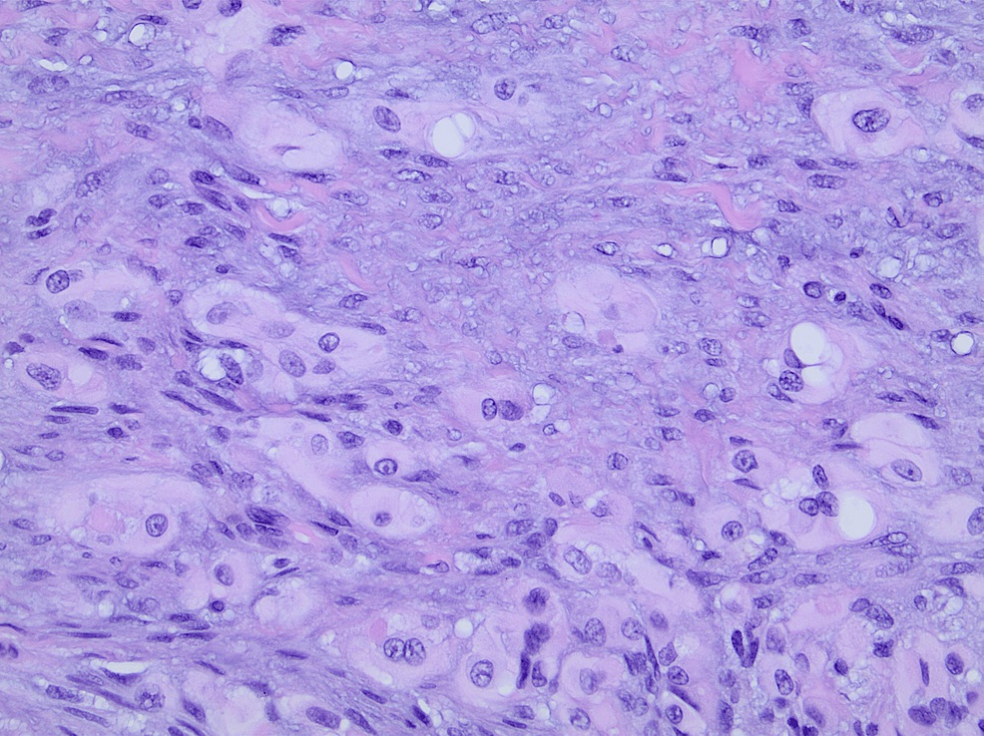
Histopathological specimen of lung showing tumor cells with intracytoplasmic vacuolization (H&E 400x). H&E, hematoxylin and eosin

A 73-year-old African-American female was followed in the pulmonary clinic for shortness of breath that was going on for 3 months. She was found to have multiple pulmonary nodules and pleural thickening that remained initially stable on serial CT imaging. She had progressive fatigue, dyspnea, cough, and pleuritic chest pain and was found to have a small left-sided pleural effusion and abnormal right paratracheal lymph nodes (1.5 cm in size) with increasing pulmonary nodules. Endobronchial ultrasound-guided transbronchial needle aspiration (EBUS-TNBA) of the right paratracheal (station 4R) lymph node was done for diagnostic purposes, but the final pathology on the cell block did not reveal any malignant or diagnostic cells. The patient experienced persistent shortness of breath and was admitted for further evaluation. Imaging (Figure 1) revealed loculated pleural effusion, plaque-like pleural nodularity extending adjacent to the pericardium, and new opacification of the left lung, which was revealed via bronchoscopy to result from extrinsic compression of the left lower lobe. Thoracentesis identified exudative effusion with normal pH, negative Gram staining, cultures, and fluid cytology negative for malignancy. The patient underwent video-assisted thoracoscopic surgery (VATS) with decortication to address the recurrent effusion and mediastinal adenopathy in the setting of a negative EBUS-TBNA. Preliminary results of a pleural biopsy were concerning for mucinous adenocarcinoma and sent for further evaluation. Final pathological evaluation of pleural peel, inferior phrenic ligament, pericardium, lymph node, and lung biopsies showed epithelioid cells with rounded nuclei, scant cytoplasm, and prominent intracytoplasmic vacuoles. A panel of immunostains was performed, and the tumor cells were positive for CD34, podoplanin, and vimentin and were negative for pancytokeratin, Cam5.2, CK7, CK20, calretinin, WT1, S100, melanoma cocktail, EMA, Gata3, p40, TTF-1, and Pax-8. These findings were consistent with a diagnosis of pleural subtype EHE (Figure 2). 

The patient returned to the clinic following her thoracotomy and decortication with symptomatic improvement, reduced need for supplemental oxygen, and scheduled outpatient follow-up with oncology where she was offered a combination of chemotherapy and radiation therapy. Her pleural effusion did not recur and she continued her follow-up with the Oncology team. 

## Discussion

Epithelioid hemangioendothelioma is a rare endothelial vascular neoplasm of soft tissue, described as an intermediate of hemangioma and angiosarcoma [1]. This neoplasm has been known to manifest in a number of tissues including the liver, lung, and soft tissue, as well as the pleura. The pleural variant is particularly uncommon and often difficult to diagnose.

The patient described in this case presented with non-specific symptoms of cough and dyspnea. Pleural effusion is a prominent feature of pleural EHE and is commonly loculated, like in this case. The pleural effusion is usually exudative due to inflammation causing increased capillary permeability. The differential diagnosis for this constellation of symptoms is broad and includes malignancies, infections, and autoimmune etiologies.

CT imaging of this patient demonstrated multiple stable pulmonary nodules, plaque-like pleural thickening, and lymphadenopathy, in addition to unilateral loculated pleural effusion. These findings are typical for pleural EHE, which most commonly features unilateral pleural fluid and nodular pleural thickening; multiple pulmonary nodules may indicate metastases, consistent with the aggressive nature of the pleural variant of this neoplasm [2].

Biopsy of the neoplastic tissue confirms the diagnosis of pleural EHE and differentiates it from other malignancies of the pleura. Histologically, EHE features epithelioid cells with abundant eosinophilic cytoplasm in a fibromyxoid stroma, which may have a signet-ring appearance due to the potential presence of intracytoplasmic vacuole. The cells of this neoplasm share characteristics with non-neoplastic endothelial cells, but are distinguished by immunochemical staining and electron microscopy [3]. Pathologic exam of pleural biopsy samples in this case follows this pattern and provides another diagnostic hurdle. The focal association of the epithelioid tumor cells with myxoid stroma simulated a mucinous neoplasm on preliminary pathology evaluation. Immunostaining of the neoplastic tissue was positive for CD34, podoplanin, and vimentin, consistent with the diagnosis of EHE of the pleura.

Treatment options for pleural EHE include a complete surgical resection, which in many cases is not possible due to metastasis and patient debility. Other options like chemotherapy with carboplatin plus etoposide have been tried with some success [4]. As for symptomatic management, the pleural effusion in EHE is mostly loculated hence treatment of symptomatic pleural effusion includes video-assisted thoracic surgery (VATS) decortication. There is not much literature on the use of indwelling pleural catheters or pleurodesis in these patients who might suffer from recurrent symptomatic pleural effusion. Aggressive pain control is important. Pleural EHE has an aggressive clinical course and the presence of pleural effusion portends to poor prognosis [5-6].

## Conclusions

The pleural variant of EHE is an aggressive neoplasm presenting with non-specific symptoms. Other than pleural effusion, it can present with pulmonary nodules and mediastinal lymphadenopathy. The diagnosis requires stepwise approach and can be delayed due to its low prevalence. Diagnosis eventually requires biopsy with immunostaining differentiating it from other pleural neoplasms.
